# Incidence rate of schizophrenia after the Tangshan earthquake in China: a 44-year retrospective birth cohort study

**DOI:** 10.1038/s41398-022-02125-3

**Published:** 2022-09-06

**Authors:** Yun-Shu Zhang, Wen-Wang Rao, Li-Li Zhang, Hong-Xue Jia, Hao Bi, Hai-Long Wang, Lloyd Balbuena, Ke-Qing Li, Yu-Tao Xiang

**Affiliations:** 1 Hebei Key Laboratory of Major Mental and Behavioral Disorders, Hebei Provincial Mental Health Center, Baoding, Hebei Province China; 2 Institute of Mental Health, Hebei Provincial Mental Health Center, Baoding, Hebei province China; 3 College of Public Health, Hebei University, Baoding, Hebei province China; 4 Department of Psychiatry, University of Saskatchewan, Saskatoon, SK Canada; 5 Unit of Psychiatry, Department of Public Health and Medicinal Administration, & Institute of Translational Medicine, Faculty of Health Sciences, University of Macau, Macao SAR, China; 6 Centre for Cognitive and Brain Sciences, University of Macau, Macao SAR, China; 7 Institute of Advanced Studies in Humanities and Social Sciences, University of Macau, Macao SAR, China

**Keywords:** Scientific community, Human behaviour

## Abstract

Preliminary evidence indicates that natural disasters are associated with an increased risk for schizophrenia. With few longitudinal studies on earthquakes, this retrospective cohort study examined exposure to the 1976 Tangshan earthquake and the subsequent risk of schizophrenia. Population counts and visits to all nine psychiatric hospitals in Tangshan city were collected. We created three cohort groups by earthquake exposure: infant (August 1972 to July 1976 births), fetal (August 1976 to May 1977 births), and unexposed (June 1977 to May 1981 births). The cumulative incidence of schizophrenia in each cohort was calculated by dividing the number of schizophrenia patients by total births in the corresponding period. Altogether, 6424 schizophrenia patients were identified, with 2786 in the infant group, 663 in the fetal group, and 2975 in the unexposed group. The crude cumulative incidence of schizophrenia in the infant, fetal and unexposed groups were 7.64 (95% confidence interval [CI] = 7.36–7.92), 9.07 (95% CI = 8.38–9.76), and 7.40 (95% CI = 7.13–7.66) per thousand population respectively. Adjusted for mortality, the corresponding figures were 7.73 (95% CI = 7.44–8.01), 9.30 (95% CI = 8.60–10.01) and 7.44 (95% CI = 7.18–7.71) per thousand population respectively. The mortality-adjusted risk ratio (aRR) was 1.25 (95% CI = 1.15–1.36) between fetal and unexposed groups (*χ*^2^ = 27.31, *P* < 0.001). Males exposed as infants did not differ from the unexposed in cumulative schizophrenia incidence. People with fetal exposure to the 1976 earthquake had 25% higher risk of developing schizophrenia compared to unexposed counterparts.

## Introduction

Schizophrenia is a severe mental illness, accounting for about 13.4 million years lived with disability according to the Global Burden of Disease study [1]. It is well-known that natural disasters increase the risk for mental health problems [2, 3]. A report from World Health Organization (WHO) estimated that approximately 20% of a given population have higher risk for later mental health problems when exposed to a disaster [4, 5].

Natural disasters (e.g., earthquakes, floods, storms, etc.) frequently lead to physical and psychological stress in the survivors [6, 7]. There is ample research showing that prenatal and perinatal stress from natural disasters affect the mental health of the offspring [8], including an increased risk for schizophrenia [9]. There is evidence that major traumatic events in pregnancy activate the hypothalamus-pituitary-adrenal (HPA) axis, which plays a key role in orchestrating bodily responses to stress [10]. HPA axis activation increases cortisol levels and glucocorticoid secretion. Numerous studies revealed that abnormal maternal hormone levels influence the development of the fetus through changes in placenta homeostasis [11–13].

To date, there are mixed findings regarding the association of schizophrenia with disasters. Early studies found no association between onset of schizophrenia or bipolar disorder and disasters [14, 15]. However, a cross-sectional study found that participants exposed to earthquakes had a higher risk of schizophrenia, compared to unexposed [9]. In addition, a national study from the second China National Sample Survey on Disability (CNSSD) reported that prenatal exposure to earthquakes is associated with the long-term risk of adult schizophrenia [16]. Although the study was well-designed, it relied on the CNSSD to identify schizophrenia cases. Since the CNSSD used a household-based sampling and excluded institutionalized patients, it may have underestimated the number of people developing schizophrenia. This is because people who do not own a home, are unmarried, or are frequent visitors to hospitals tend to be underrepresented in population surveys [17, 18].

In this study, we compared cumulative incidence rates of schizophrenia in people with varying earthquake exposure in order to test the fetal origins hypothesis. Based on previous findings [9, 16], we hypothesized that the incidence rate of schizophrenia among individuals exposed to the 1976 earthquake would be higher than in the unexposed.

## Methods

### Subjects

This is a retrospective cohort study of schizophrenia risk in Tangshan City, in the eastern part of Hebei Province. On July 28, 1976, Tangshan City was devastated by a magnitude 7.8 earthquake that killed approximately 250,000 people [19]. Tangshan City had a population of approximately 7.4–7.9 million during the period between 2015 and 2020 [20, 21]. Table 1 presents descriptive statistics of Tangshan’s population circa 1976: note the negative growth rate for that year. Nine psychiatric hospitals (Tangshan Mental Health Center, Kailuan Mental Health Center, Qian’an Mental Health Center, Tangshan Psychiatric Hospital, Luan’nan Mental Health Center, Yutian Psychological Rehabilitation Center, Fengrun Aixin Psychiatric Rehabilitation Center, Leting Xiangting Hospital, and Zunhua Sujiawa Health Center) provide mental health services for Tangshan city residents.

**Table 1:** Total population and birth, death, and natural growth rate per 1000 residents during 1972 and 1981 in Tangshan city.

Year	Total population	Birth rate	Birth population	Death rate	Natural growth rate
1972	5,406,186	23.0	124,342	7.4	15.6
1973	5,475,588	18.7	102,393	6.1	12.6
1974	5,520,995	14.5	80,054	6.7	7.8
1975	5,569.355	14.7	81,870	7.0	7.7
1976	5,407,751	14.9	80,575	44.1	−29.2
1977	5,487,908	16.8	92,197	6.4	10.4
1978	5,584,723	18.6	103,876	5.9	12.7
1979	5,650,905	17.8	100,586	5.9	11.9
1980	5,730,658	16.6	95,129	6.1	10.5
1981	5,821,476	20.3	118,176	5.9	14.4

The cohort consisted of people born between August 1972 and May 1981 in Tangshan City. From this cohort, three groups were formed: infant exposure (born between August 1972 and July 1976), fetal exposure (born between August 1976 and May 1977), and unexposed (born between June 1977 and May 1981). The study protocol was approved by the Ethics Committee of all participating centers/hospitals. Informed consent was waived due to the retrospective design of this study.

### Data collection and measurements

Population data from 1972 to 1981 were obtained from the Tangshan Statistical Yearbook in 1973–1982 and the 1982 Population Census of China [22]. A two-stage procedure was followed to identify people who developed schizophrenia after the 1976 earthquake. In stage A, following previous studies [23, 24], all psychiatric referrals were retrieved from the electronic record systems of the abovementioned nine psychiatric hospitals. This data covered both inpatients and outpatients who were born between 1972 and 1981, and from these records, patients diagnosed with schizophrenia were extracted. Following a previous study [24], all cases with a diagnosis of schizophrenia based on the Chinese Classification of Mental Disorders (CCMD), ICD-9 or ICD-10 between 1990 and 2020 (end of data collection) were selected. For those with a CCMD or ICD-9 diagnosis of schizophrenia, the diagnosis of schizophrenia was validated according to the International Classification of Diseases, Tenth Revision (ICD-10) criteria [25] by trained research psychiatrists in Hebei Provincial Mental Health Center based on a review of medical records. The diagnostic criteria for schizophrenia in the CCMD are very similar to the ICD-10 with high diagnostic concordance [26]. We further examined a national community‐based mental health management system (i.e., the “686 program”) in stage B to capture patients that may have been missed previously. The “686 program” was set up in China to effectively manage millions of community-dwelling patients with severe psychiatric disorders [27, 28]. Trained investigators searched for target patients living in Tangshan city according to their date of birth.

Basic socio-demographic and clinical characteristics of the patients were collected by reviewing medical records. This was carried out by trained research assistants who were blinded to the study protocol. The variables of interest included date of birth, sex, ethnicity, education level, marriage status, employment status, and having a family history of psychiatric disorders. Age of illness onset was indexed to the first contact with psychiatric services. To ensure the accuracy and reliability of data collection, a pre-study workshop was organized to agree on standard procedures. An inter-rater reliability exercise was performed, and kappa values greater than 0.8 for a schizophrenia diagnosis were obtained between research assistants. Any discrepancy in diagnosis between research psychiatrists were resolved by an expert panel established for this study. Raw data that were first recorded in data collection sheets were then entered into Epi data software (version 3.1, Odense, Denmark).

### Statistical analysis

The cumulative incidence of schizophrenia was calculated for people with infant exposure, fetal exposure, and unexposed. First, the crude cumulative incidence schizophrenia was calculated by dividing the number of births in each group who subsequently developed schizophrenia by the total births in the same period. This crude cumulative incidence was then adjusted for mortality by removing deaths from the denominator (i.e., total births minus total deaths) but leaving the numerator as is. This was done separately for each group. The average annual schizophrenia incidence was estimated based on the cumulative incidence rate divided by average follow-up period (years since age 18). We then made group comparisons by calculating crude risk ratios (RRs) and mortality-adjusted risk ratios (aRRs) of schizophrenia incidence. Please refer to Table 4 for the exact formulas used in the calculation. A hierarchical Chi-Square test was used to examine sex differences in schizophrenia risk between the infant group, the fetal group, and the unexposed group. These calculations were implemented in SPSS v. 26 (IBM SPSS, IBM Corp., Armonk, NY, USA).

## Results

### Evidence of earthquake from birth rates and mortality rates

**Fig. 1:**
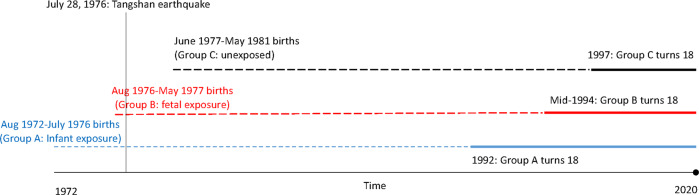
Timeline of cohort births by exposure to the Tangshan earthquake. Dashed lines represent the period in which cohort members could not be diagnosed with schizophrenia. Solid lines represent the follow-up time in which cohort members could be diagnosed with schizophrenia.

Table 1 also summarizes the birth and death rates at Tangshan city. Although birth rates were relatively stable between 1972 and 1981, the mortality rate spiked in 1976, leading to a negative growth rate, reflecting lives lost to the earthquake. Figure 1 presents the three cohorts relative to the earthquake and the years in which they turned 18.

### Increased risk of schizophrenia

Table 2 presents demographic and clinical characteristics of schizophrenia patients by birth group. Between August 1972 and May 1981, 2786 people in the infant group later developed schizophrenia, 663 in the fetal group, and 2795 in the unexposed. These schizophrenia numbers correspond to the following period births: 364,718 in the infant group, 73,105 in the fetal group, and 402,103 in the unexposed.

**Table 2:** Basic demographic and clinical information of schizophrenia patients.

Group	Birth year	No. of schizophrenia cases	No. (%) of men	No. (%) of Han	Education level I	Education level II	No. (%) of married	No. (%) of employed	Age of onset	No. (%) of familial cases^a^
Infant exposure group	August, 1972– July, 1976	2786	1233 (44.3)	2733 (98.1)	979 (35.1)	1588 (57.0)	1482 (53.2)	2030 (72.9)	27.03 ± 7.14	422 (15.1)
Fetal exposure group	August, 1976– May, 1977	663	322 (48.6)	650 (98.0)	232 (35.0)	374 (56.4)	369 (55.7)	450 (67.9)	25.85 ± 6.65	98 (14.8)
Unexposed group	June, 1977– May, 1981	2975	1429 (48.0)	2906 (97.7)	937 (31.5)	1770 (59.5)	1837 (61.7)	2031 (68.3)	25.54 ± 6.31	454 (15.3)

Note: Education Level I = Primary school or below; Education Level II = Secondary school.

^a^Having a positive family history of psychiatric disorders in schizophrenia patients.

Table 3 is a comparison of relative risks for schizophrenia across groups. The cumulative incidence of schizophrenia in the infant, fetus, and unexposed groups were 7.64 (95% confidence interval [CI] = 7.36–7.92), 9.07 (95% CI = 8.38–9.76) and 7.40 (95% CI = 7.13–7.66) per thousand population, respectively. When adjusted for mortality, the cumulative incidence was 7.73 (95% CI = 7.44–8.01), 9.30 (95% CI = 8.60–10.01) and 7.44 per thousand population (95% CI = 7.18–7.71) in infant, fetus, and unexposed groups, respectively. Compared to the unexposed group, the adjusted risk ratio for schizophrenia in the fetal group was higher (aRR: 1.25; 95%CI = 1.15–1.36). There was no significant difference between the infant and unexposed groups (aRR: 1.04; 95%CI = 0.99–1.09). The crude annual incidence of schizophrenia was 27.3, 35.6, and 32.2 per 100,000 population in the infant, fetal and unexposed groups, respectively, indexed to the years of follow-up from age 18 (Table 4).

**Table 3:** Risk of developing schizophrenia in people born between August 1972 and May 1981 in Tangshan City.

Group	Year	Cases	No. of births	No. of mortality	Unadjusted incidence, ‰	Mortality-adjusted Incidence, ‰	Unadjusted RR (95%CI)	Adjusted RR (95%CI)	*χ*^2^	*P* value
Infant exposure group	August,1972– July,1976	2786	364,718	4093	7.64	7.73	1.03 (0.98–1.09)	1.04 (0.99–1.09)	2.01	0.16
Fetal exposure group	August,1976– May,1977	663	73,105	1844	9.07	9.30	1.23 (1.13–1.33)	1.25 (1.15–1.36)	27.31	<0.001
Unexposed group	June,1977– May,1981	2975	402,103	2419	7.40	7.44	1	1	-	-

**Table 4:** Calculation of the average annual schizophrenia incidence by exposure group.

Group	A: No. of schizophrenia cases	B: Total births	C: Average follow-up period (years since age 18)^a^	D: Cumulative Incidence per 100,000^b^	E: Average Incidence Rate per year of follow-up from age 18^c^
Infant Exposure (August, 1972– July, 1976)	2786	364,718	28	764	27.3
Fetal Exposure (August, 1976– May, 1977)	663	73,105	25.5	907	35.6
Unexposed Group (June, 1977– May, 1981)	2975	402,103	23	740	32.2

^a^Calculated as 2020 – year turned 18 for the median year of each cohort. Median years are 1974, 1976.5, and 1979 for the infant, fetal, and unexposed groups respectively.

^b^Calculated as Column A/Column B × 100,000.

^c^Calculated as Column D/Column C.

### Increased risk of schizophrenia by sex

#### Fetal group vs unexposed group

In men, a significantly increased risk was found (RR:1.27; 95% CI = 1.12–1.43). Similarly, there was an increased risk in women (RR: 1.24; 95% CI = 1.10–1.39). The odds ratios did not differ significantly by sex based on the test of homogeneity. (*P* = 0.765).

#### Infant group vs unexposed group

There was no significant risk difference in men (RR: 0.959; 95% CI = 0.888–1.04), but there was a significant difference in women (RR: 1.11; 95% CI = 1.04–1.19). The odds ratios differed significantly by sex based on the test of homogeneity (*P* = 0.006).

## Discussion

The present study linked hospital and community health records around the time of the Tangshan 1976 earthquake to determine if the cumulative incidence of schizophrenia differed by exposure. We found that people who were in gestation had significantly higher risk of schizophrenia compared to the unexposed group. There was no significant difference between the infant and unexposed group.

The mortality-adjusted cumulative incidence of schizophrenia in our study were higher than the corresponding numbers in the study by Guo and their colleagues [16]. While they used a complex survey design, we made use of administrative records of births, deaths, and psychiatric diagnoses. A systematic review and meta-analysis of one hundred studies found that the median incidence of schizophrenia for every 100,000 persons in the general population was 15.2 (80 percent CI = 7.7–43.0) [29]. By comparison, the crude annual incidence of schizophrenia in the infant, fetal, and unexposed groups in the present study were 27.3, 35.6, and 32.2 respectively. The spike in the fetal group can probably be attributed to the earthquake [30] and is consistent with previous findings [7, 9].

This study found that female infants had an increased risk of schizophrenia, but not male infants. An earlier study also found that women were more likely to develop post-traumatic stress disorder (PTSD) compared to men [31], which was associated with higher risk of subsequent schizophrenia [32]. Similarly, previous studies also found that women are more vulnerable to stress/disaster, compared to men [33]. It would be important to study this sex difference in future research.

Our findings support the fetal origins hypothesis. From a developmental perspective, the brain’s structure and function can be influenced by events in gestation [34]. For example, maternal infections were associated with schizophrenia in the offspring in adulthood [35]. People exposed to a 6.1 magnitude earthquake in Italy exhibited HPA axis activation for up to a year after the event [36]. Maternal stress resulting from the Tangshan earthquake may have induced neurobiological changes in the fetus, thereby increasing the risk for schizophrenia [37]. Without detailed individual-level data, we were unable to examine the transmission of trauma from mother to child. There is some evidence from studies with parent-child cohorts that risk can be passed across generations [38–41]. Additionally, some mechanisms proposed for intergenerational transmission include epigenetic changes and poor parenting skills among those exposed to trauma [40, 42].

We acknowledge several methodological limitations of this study. Despite our best efforts, the incidence rate may still be underestimated. Schizophrenia patients or their families may not have sought treatment, because of the social stigma in doing so. Mental illness causes a “loss of face” in China, but this was more pronounced in the 1970’s [43]. Furthermore, we did not have serological measures of maternal stress that could serve as a basis for comparing exposed and unexposed groups. Similar to previous findings [23, 24, 44], the lack of person-level microdata precluded us from carrying out a multivariate analysis, so the possibility that various confounders accounted for the elevated incidence in the fetal group cannot be discounted. Due to the lack of population data at the weekly level, the relationships between periods of pregnancy and the risk of schizophrenia were not examined. Finally, relevant data on the incidence rate of schizophrenia in 1, 2, and 3 years after the earthquake were not available.

## Conclusions

The findings provided evidence that people with fetal exposure to the 1976 earthquake had 25% higher risk of developing schizophrenia compared to unexposed counterparts. Although the Tangshan earthquake occurred more than four decades ago, many who experienced it survived to the present. It would be important for the health system to continue monitoring the mental health of middle-aged survivors—whether they have developed schizophrenia or not. Laying the foundation for interventions addressing the acute and long-term needs of disaster survivors is important.
